# Sudden onset methaemoglobinaemia in a previously well Ugandan child: a case report and literature review

**Published:** 2012-03-19

**Authors:** Nicolette Nabukeera-Barungi, Edison Mworozi

**Affiliations:** 1 Department of Paediatrics and Child Health, Makerere University, College of Health Sciences and Mulago National Referral Hospital, Kampala, Uganda

**Keywords:** Methaemoglobinaemia, intoxication, nitrites, food preservatives, Hemoglobine, Uganda

## Abstract

Methaemoglobinaemia is a rare condition of unknown prevalence. Diagnostic tests in resource limited settings are very rare but clinical signs can be a good guide. We set out to describe a case of Methaemoglobinaemia, raise awareness among practitioners in resource limited settings and to share experiences in its diagnosis and management. A previously well three and a half year old girl was admitted with central cyanosis of sudden onset. She underwent clinical, laboratory and radiological evaluation. Having been in a resource limited setting, the process of making a diagnosis was slow and difficult. After the diagnosis, the treatment was not available in the country but we managed to get it all the way from Nairobi, Kenya. A diagnosis of Methaemoglobinaemia was made using Spectrophotometry and she was successfully treated using 2 doses of intravenous Methylene blue. The cause of Methaemoglobinaemia was established to have been nitrites from food preservatives.

## Introduction

Methaemoglobinaemia (MetHba) is a rare condition. There is scarce data on this condition in Africa. We set out to describe a case of MetHba following nitrite ingestion through food preservatives. In this case report, we highlight the difficulties in managing the case in a resource limited setting like a public Hospital in Kampala, Uganda.

## Patient and case report

A 3 and a half year old previously well girl was referred to Hospital with a diagnosis of unexplained cyanosis. The child had been well until 9 pm on the day of admission when the mother, a medical doctor, all of a sudden noticed that the child’s lips were blue. She was not a known cardiac patient and did not have any cough or difficulty in breathing. However, she had eaten food from a fast food restaurant 6 hours earlier and had vomited 2 hours later but remained generally well. She did not have diarrhea, abdominal pain, fever or loss of appetite and she was playing. They reported no history suggestive of poisoning although there was some rat poison which was in easy reach of the child. The younger brother who ate the same food was completely well except that the following day he passed loose stool twice. Initial physical examination at the Hospital revealed a child in good general condition, well nourished, alert and playing. She had central cyanosis, was afebrile, not in respiratory distress and was not anaemic. She had equal air entry bilaterally and normal breath sounds. In the Cardiovascular system, the pulse rate was 150 per minute, normal volume and heart sounds were normal. Notably, the motor exam was normal. Oxygen saturation by pulse oxymetry was 80% without oxygen. Other systems were normal.

Full blood Count done was normal, with an Hb of 12.6g/dl, total white blood cell count was 7,700/ml. The child was given only Oxygen therapy at 2 liters. While on Oxygen, the saturation only raised to 85%. Chest X-Ray, ECG and Echocardiography were done and they were all normal. At that time, we made a diagnosis of Methaemoglobinaemia following poisoning, and thought it was so mild that it would resolve spontaneously on only Oxygen. On day 3, while still on oxygen, the child’s condition deteriorated, with saturations going down to 68%. She became so weak that she could not stand unsupported, was restless and irritable and even got visual hallucinations. She lost appetite and vomited twice. She however remained conscious. We concluded that the mental confusion was due to the hypoxia or toxins from the poison and intervention was needed. Methaemoglobinaemia needed to be treated but there was neither a way of confirming it nor drugs to treat it at that time. By the end of the day, we had found out where to do the toxicology screen and also confirm Methaemoglobinaemia using Spectrometry. On Day 4, blood samples were taken to the Government Analytical Laboratory and the diagnosis of Methaemoglobinaemia was confirmed. Toxicology screen of blood and urine revealed Nitrites in both. After confirming the diagnosis, Methylene Blue was flown in from Nairobi, Kenya within 12 hours. The child was taken to Intensive Care Unit and given high flow Oxygen up to 5 liters and Ascorbic acid as we awaited arrival of Methylene Blue. The general condition improved remarkably but she remained cyanosed. Cyanosis stopped after 2 doses of intravenous Methylene Blue at 30mg per dose, 6 hours apart. Oxygen was gradually reduced titrating against the saturation. She was in Hospital for 9 days in all and went home fully recovered.

## Discussion

Methemoglobinemia (MetHba) is a clinical syndrome caused by an increase in the blood levels of methemoglobin (MetHb) [1]. MetHb is the oxidized form of haemoglobin and incapable of carrying oxygen. The concentration of MetHb does not exceed 1%–2% in the normal physiological state. Increased levels of MetHb lead to tissue hypoxia and can be fatal. Its prevalence is difficult to determine because it encompasses a wide spectrum ranging from mild cases, which are probably under diagnosed to fatal cases [1].

Aetiology of MetHba is secondary to either toxic agents or it may be as a result of congenital enzyme deficiencies [1,2]. Toxic causes include drugs like Benzocaine, Chlorates, Chloroquine, Dapsone, Nitrophenol, Phenazopyridine, phenacetin derivatives, Primaquine, Sodium nitroprusside, 4-dimethylaminophenol, aniline dyes, sulphonamides and quinones. Exposure to nitrites and nitrate compounds (for example, amyl nitrite, glyceryl trinitrite) is the commonest cause of acquired Methaemoglobinaemia [3,4]. Those drugs are powerful oxidants widely used as preservatives and dyes in the food industry [4,5]. They can be found in industrialized baby foods, barbecue-flavored foodstuffs, and other products [2,3]. Nitrates can also be present, as a contaminant, in drinking water. Local anesthetics (benzocaine, lidocaine, and prilocaine) are also common causes. Intoxication with pesticides, herbicides, and fertilizers, automobile exhaust fumes, and industrial chemicals has also been associated with MetHba. [2]. This child ingested Nitrites from food preservatives.

In the pathophysiology of Metha, each Hb molecule has four atoms of iron. Each ferrous iron can reversibly link one O_2_ molecule, for a total of four molecules of O_2_ transported by each Hb molecule [1,3]. Methemoglobin is the oxidized form of Hb, whose heme Fe^+2^ is oxidized to ferric iron (Fe^+3^) and for this reason, cannot bind oxygen. Ferric iron also causes an allosteric change in the heme portion of partially oxidized Hb, increasing its O2 affinity. Thus, besides the inability to bind O2, MetHb shifts the dissociation curve of partially oxidized Hb to the left, hindering the release of O2 in the tissues [1,3,4]. Tissue hypoxia caused by MetHba is secondary to a reduction in free Hb to transport O2 and the difficulty to release O2 in the tissues.

Clinical manifestations of MetHba reflect the reduction in O2-carrying capacity, leading to tissue hypoxia [5]. Clinical features depend on the level of Methaemoglobinaemia. One should suspect MetHba in patients with central cyanosis and low saturation on pulse oxymetry refractory to oxygen administration once more common causes, like cardiopulmonary dysfunctions, are ruled out [5]. This was the presentation of this child, and the diagnosis was entertained after the tests showed normal cardiac and respiratory functions. Below 3%, there are no signs and symptoms. Between 3-15%, there are frequently no symptoms but they may present with grayish skin color. Cyanosis starts above 15% and it does not respond to Oxygen administration [1,2,6]. They also have chocolate brown blood between 15-30%. Dyspnoea usually sets in between 30-50%. At this level, the SpO2 is about 85%. Dizziness, fatigue, headache, weakness and syncope may also be present at this level. Severe forms present with metabolic acidosis, cardiac arrhythmias, seizures, CNS depression and coma when levels are 50-70%. Death usually occurs with levels about 70% [1–3]. In this patient, the levels of MetHba were not measured but from the clinical presentation, she should have had between 30-50% since she had weakness and SpO2 below 85%. The severity of the case depends on the amount of toxin the patient has been exposed to, individual metabolic capacity, intestinal absorption, and entero-hepatic circulation. This could explain why the younger sibling who ate the same food did not develop MetHba.

Co-oximetry is the gold standard for the diagnosis of MetHba [1]. The co-oximeter is capable of measuring the concentration of different types of Hb in the blood through spectrophotometry, using different wavelengths. On measuring the arterial blood gases, the p02 is normal while the measured oxygen saturation is decreased [4]. In this child, Spectrophotometry was done and it revealed Methaemoglobinaemia. Toxicology screen revealed Nitrites as a cause.

Treatment of patients with MetHba should be guided by the severity of the disorder [2]. General measures include provision of High flow oxygen and identification of offending agent and prevention of further exposure. These measures alone are usually adequate for mild cases. Treatment with Methylene Blue (MB) is advised when the MetHb level is >30%–40% but each case must be treated individually on clinical grounds and symptoms. In situations of significant clinical manifestations (e.g., dizziness, headache, anxiety, dyspnea, symptoms of low cardiac output, somnolence, and seizures), besides the basic conduct mentioned, MB should be used as a specific antidote. Several authors suggest that MB should be used with MetHb above 30%, regardless of the presence or absence of symptoms [2,5].

Methylene blue is a thiazine dye with dose-dependent antiseptic and oxidizing properties [1]. During its use, it activates NADPH methemoglobin reductase, which reduces methylene blue to methylene leucoblue, which transforms MetHb in HHb by a non-enzymatic mechanism. The recommended dose is 1–2 mg/kg given intravenously over five minutes. Additional doses can be administered every hour if necessary, up to a maximum total dose of 7 mg/kg. In G6PD deficiency, red blood cells do not produce enough NADPH to reduce methylene blue to methylene leucoblue; N-acetyl-cysteine has instead been used on those cases [1]. In our patient, G6PD deficiency was negative and she responded well to MB. Another treatment for MetHba is ascorbic acid, but acquired MetHba does not respond to it because its capacity to reduce MetHb is much inferior to that of endogenous enzymatic systems [1]. Exchange transfusion is reserved for severe cases that do not respond to MB [1,2]. Though congenital MetHba is usually benign, the form due to a defective reducing system can be treated with ascorbic acid taken daily. The other congenital form due to hemoglobin M has no treatment as of late [1].

## Conclusion

Our case report demonstrated that MetHba can be diagnosed and successfully treated in a resource limited setting. It also showed that MetHba can present acutely in a previously well child following ingestion of nitrites from food preservatives. Practitioners in resource limited settings could draw from this experience and suspect MetHa in any cyanosed child without respiratory or cardiac problems.
